# Clopidogrel, an ADP‐P2Y12 Receptor Antagonist, Is Associated With Lower Opioid Consumption and a Reduced Incidence of Delirium in Cancer Patients Receiving Chemotherapy: A Nationwide Retrospective Cohort Study in Japan

**DOI:** 10.1002/npr2.70142

**Published:** 2026-06-14

**Authors:** Hiroaki Abe, Reo Inoue, Takamichi Kogure, Rikuhei Tsuchida, Hiroaki Owada, Yoshika Sudo, Yaeko Yokoshima, Hiroki Matsui, Hideo Yasunaga, Masahiko Sumitani

**Affiliations:** ^1^ Department of Pain and Palliative Medicine The University of Tokyo Hospital Tokyo Japan; ^2^ Department of Anesthesiology and Pain Relief Center The University of Tokyo Hospital Tokyo Japan; ^3^ Department of Clinical Epidemiology and Health Economics, School of Public Health The University of Tokyo Tokyo Japan

**Keywords:** cancer pain, clopidogrel, drug repositioning, opioids, real‐world data

## Abstract

**Background:**

P2Y12 receptor activation stimulates spinal microglia and exacerbates pain. However, whether clopidogrel, a P2Y12 receptor antagonist, has analgesic properties remains unclear. This study aimed to evaluate the association between clopidogrel use and opioid consumption in cancer patients receiving chemotherapy.

**Methods:**

We identified cancer patients receiving inpatient chemotherapy between January 2019 and December 2020 using the Diagnosis Procedure Combination database, a Japanese national inpatient database. Propensity score matching was conducted between clopidogrel and aspirin users. The primary outcome was opioid consumption during hospitalization. The secondary outcomes included analgesic use (opioids, acetaminophen, nonsteroidal anti‐inflammatory drugs, and gabapentinoids) and hyperactive delirium.

**Results:**

Among 71 554 eligible patients (male, 73%; mean age, 73.7 years), 25.3% were administered clopidogrel and 74.7% were administered aspirin. Propensity score matching included 18 135 and 36 270 patients in the clopidogrel and aspirin groups, respectively. The median daily opioid consumption in morphine milligram equivalent was significantly lower in the clopidogrel group than in the aspirin group (16.4 mg/day vs. 21.4 mg/day; *p* < 0.001). The analgesic use was significantly lower in the clopidogrel group for opioids (odds ratio [OR], 0.83; 95% confidence interval [CI], 0.78–0.88; *p* < 0.001), acetaminophen (OR, 0.91; 95% CI, 0.88–0.95; *p* < 0.001), and nonsteroidal anti‐inflammatory drugs (OR, 0.83; 95% CI, 0.80–0.87; *p* < 0.001). Moreover, the occurrence of hyperactive delirium was significantly lower in the clopidogrel group (OR, 0.80; 95% CI, 0.69–0.92; *p* = 0.002).

**Conclusions:**

Clopidogrel may have an analgesic effect in cancer patients receiving chemotherapy.

## Abstract 2

Clopidogrel use was associated with reduced opioid consumption, lower use of other analgesics, and a decreased incidence of hyperactive delirium compared with aspirin in cancer patients receiving chemotherapy, suggesting a potential analgesic effect possibly linked to P2Y12 receptor inhibition.

## Graphical abstract

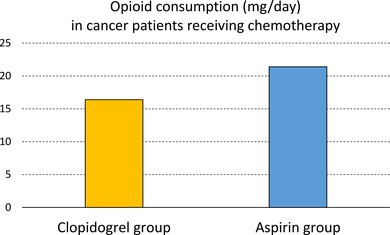

## 1 Introduction

Spinal microglia play an important role in pain mechanisms [1]. Microglia, the resident immune cells of the central nervous system, are known to contribute to both the initiation and maintenance of pain. When activated, these cells release various pro‐inflammatory mediators that sensitize neurones and amplify nociceptive signaling [1].

The purinergic P2Y12 receptor expressed in microglia is a critical modulator of their activity. Activated by extracellular adenosine diphosphate, P2Y12 receptors mediate microglial recruitment and the release of pro‐inflammatory mediators, contributing to pain transmission [2]. Studies involving animal models have shown that inhibiting P2Y12 receptors reduces pain behaviors, highlighting their role in pain pathophysiology [3].

Clopidogrel, a selective antagonist of P2Y12 receptors, may suppress microglial activation and the subsequent pro‐nociceptive cascade by inhibiting P2Y12 receptor activity. This hypothesis is supported by preclinical studies indicating that clopidogrel reduces microglial activation and alleviates pain behaviors in rodent models [4]. However, clinical evidence regarding the analgesic effects of clopidogrel remains limited.

We previously reported that genetic polymorphisms of the P2Y12 receptor are considered potential key regulators in mitigating the exacerbation of both postoperative and cancer pain [5]. In a retrospective chart review with a small‐sample size, we also found that patients who regularly used clopidogrel required less opioid medication for postoperative pain management compared to those taking aspirin [6]. These findings suggest that P2Y12 receptor antagonists could potentially serve as adjunctive therapies in pain management.

Although clopidogrel is primarily used to prevent thrombotic events in cardiovascular diseases, it offers the opportunity to explore its potential therapeutic effects beyond its primary application in cardiovascular diseases. This study investigated the relationship between clopidogrel use and opioid consumption in cancer patients receiving chemotherapy. By examining this association, we aimed to evaluate the potential analgesic benefits of P2Y12 receptor antagonists and contribute to the development of new pain management strategies.

## 2 Methods

### 2.1 Setting and Data

This study followed the Strengthening the Reporting of Observational Studies in Epidemiology guidelines [7]. It was approved by the Institutional Review Board and Ethics Committee of The University of Tokyo. Due to the anonymised nature of the data, the requirement for obtaining written informed consent was waived by the Institutional Review Board.

This study utilized data from the Diagnosis Procedure Combination database, a nationwide repository of administrative claims and discharge summaries from hospitals across Japan [8]. Participation in the database is mandatory for all 82 academic hospitals in Japan but optional for community hospitals. The database includes the records of approximately 7 million inpatients from over 1000 hospitals, accounting for nearly 50% of all acute‐care hospitalisations in Japan. It comprises comprehensive information such as hospital details (anonymised hospital identifier, hospital volume, and hospital type), patient characteristics (anonymised patient identifier, postal code, sex, height, weight, and date of birth), hospitalization details (admission date, planned or emergency hospitalization, primary diagnosis, activities of daily living at admission, comorbidities upon admission, presence of dementia, and complications during hospitalization), discharge details (discharge date and discharge status), and treatment records (medication dosages and dates, procedures, surgeries, and anesthesia). Diagnoses were documented using the International Classification of Diseases, 10th Revision (ICD‐10) codes in conjunction with the Japanese text.

### 2.2 Population

Cancer patents aged 20 years or older who underwent inpatient chemotherapy between 1 January 2019 and 31 December 2020 were selected from the database. Those with schizophrenia were excluded from the study because of their frequent use of antipsychotic medications, which can interfere with the detection of delirium onset. Schizophrenia was identified as a comorbidity upon admission based on ICD‐10 codes F20.x–F29.x.

### 2.3 Exposure

Patients who used clopidogrel or aspirin within 2 days of admission were included in the clopidogrel and aspirin groups, respectively. The two‐day window was chosen to avoid missing patients who had already taken the antiplatelets in the morning on the day of admission. Those administered both clopidogrel and aspirin were excluded.

### 2.4 Outcomes

The primary outcome was the median daily opioid consumption in morphine milligram equivalents. The secondary outcomes included the use of analgesics (opioids, acetaminophen, nonsteroidal anti‐inflammatory drugs [NSAIDs] other than aspirin, and gabapentinoids) and the occurrence of hyperactive delirium within 14 days of admission. Hyperactive delirium was identified based on the initiation of antipsychotics (haloperidol or risperidone) 2 days after admission. This approach for identifying delirium was chosen because a similar algorithm demonstrated sufficient validity for use in claims‐based databases [9].

### 2.5 Potential Confounding Variables

Potential confounders included demographic factors, pre‐existing comorbidities, and concurrent medications. Variables were chosen based on factors that could influence the selection of antiplatelet agents and patient outcomes, according to clinical expertise and prior research [10, 11, 12, 13]. The demographic characteristics included sex, age, body mass index, smoking status, Barthel Index scores, hospital type (academic or non‐academic), and admission year. The Barthel Index is a standardized measure used to evaluate functional independence in activities of daily living, with higher scores reflecting greater independence [14]. Comorbidities were assessed using the Charlson Comorbidity Index scores. Additional factors, such as emergency hospitalization, vascular diseases, and cancer types, were also included. The Charlson Comorbidity Index, which quantifies the burden of comorbid conditions, was calculated using ICD‐10 codes based on Quan's algorithm [15, 16]. Vascular diseases such as coronary artery disease, cerebrovascular disease, and peripheral artery disease were included to examine the potential indications for antiplatelet use. The cancer types included commonly observed malignancies, such as lung cancer, upper gastrointestinal cancer, lower gastrointestinal cancer, and hematopoietic tumors. The medications included anticoagulants and antibiotics. For patients with missing data on body mass index, smoking status, Barthel index, or Charlson Comorbidity Index scores, separate categories were created to account for the missing values.

### 2.6 Statistical Analysis

Propensity score‐matched analysis was conducted to adjust for confounding by indication and potential baseline differences between the clopidogrel and aspirin groups [17]. Propensity scores were calculated using a multivariable logistic regression model, with clopidogrel use as the dependent variable. The independent variables included 18 potential confounders, as described earlier. Nearest neighbor matching with a one‐to‐two ratio was used for propensity score matching, with patients in the clopidogrel group matched to those in the aspirin group based on estimated propensity scores within a 0.2 standard deviation caliper width of the logit. The balance of variables between the two groups was assessed using absolute standardized differences, with a value > 0.1 indicating an imbalance [17].

Median values and interquartile ranges were used to compare daily opioid consumption between the groups, and the Mann–Whitney *U* test was used for statistical comparisons. To compare analgesic use and delirium occurrence between the groups, odds ratios (ORs) and their 95% confidence intervals (CIs) were calculated using a multivariable logistic regression model fitted with generalized estimating equations to account for within‐hospital correlations.

All reported *p*‐values were two‐sided, and *p*‐values of < 0.05 were considered statistically significant. All statistical analyses were conducted using the Stata/SE software (version 18.0; Stata Corp., College Station, TX, USA).

## 3 Results

**FIGURE 1:**
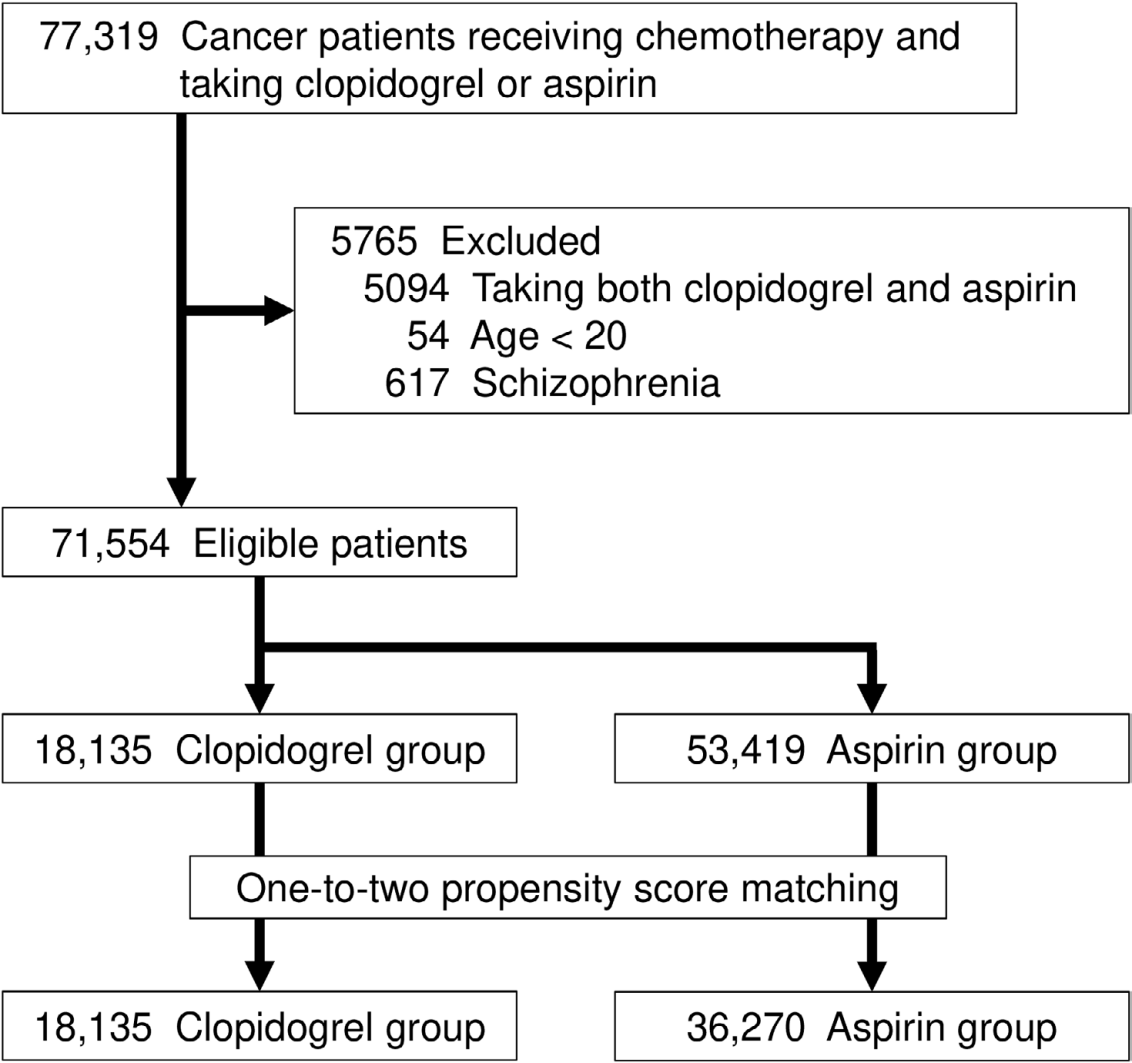
Flow chart regarding the inclusion and exclusion of patients in the study population.

We identified 77 319 cancer patients receiving inpatient chemotherapy and taking clopidogrel or aspirin. After exclusion, 71 554 patients were included (52 312 [73%] men; mean age [standard deviation], 73.7 [8.7] years; median length of stay [interquartile range], 11 [5–21] days) (Figure 1).

Table 1 presents the baseline characteristics of the unmatched and propensity‐score matched groups. One‐to‐two propensity score matching was used to create a final study cohort of 18 135 and 36 270 patients in the clopidogrel and aspirin groups, respectively. After matching, all absolute standardized differences were < 0.1, and the quality of matching was acceptable (Table 1). Table 2 presents the primary outcomes of the matched groups. The median daily opioid consumption in morphine milligram equivalents was 16.4 mg (interquartile range, 5.0–41.5) in the clopidogrel group and 21.4 mg (6.0–61.4) in the aspirin group. There was a significant difference in the daily opioid consumption between the two groups (Mann–Whitney *U* test, *p* < 0.001). Table 3 presents the secondary outcomes of the matched groups. Analgesic use was significantly lower in the clopidogrel group compared with the aspirin group for opioids (OR, 0.83; 95% CI, 0.78–0.88; *p* < 0.001), acetaminophen (OR, 0.91; 95% CI, 0.88–0.95; *p* < 0.001), and NSAIDs (OR, 0.83; 95% CI, 0.80–0.87; *p* < 0.001). The occurrence of 14‐day delirium was also significantly lower in the clopidogrel group (OR, 0.80; 95% CI, 0.69–0.92; *p* = 0.002). The use of gabapentinoids was not significantly different between the groups (OR, 0.95; 95% CI, 0.89–1.01; *p* = 0.111).

**TABLE 1:** Baseline patient characteristics in the unmatched and matched groups.

	Unmatched groups			Matched groups		
	Clopidogrel	Aspirin	ASD^a^	Clopidogrel	Aspirin	ASD^a^
	18 135	53 419		18 135	36 270	
**Demographic characteristics**						
Sex						
Male	13 898 (76.6)	38 414 (71.9)	0.11	13 898 (76.6)	28 206 (77.8)	0.03
Female	4237 (23.4)	15 005 (28.1)		4237 (23.4)	8064 (22.2)	
Age						
20–59	728 (4.0)	3364 (6.3)	0.12	728 (4.0)	1266 (3.5)	0.03
60–69	3554 (19.6)	11 525 (21.6)		3554 (19.6)	7211 (19.9)	
70–79	9172 (50.6)	25 364 (47.5)		9172 (50.6)	18 397 (50.7)	
≥ 80	4681 (25.8)	13 166 (24.6)		4681 (25.8)	9396 (25.9)	
Body mass index						
≤ 18.4	2155 (11.9)	5934 (11.1)	0.06	2155 (11.9)	4080 (11.2)	0.03
18.5–24.9	11 973 (66.0)	34 400 (64.4)		11 973 (66.0)	24 378 (67.2)	
≥ 25.0	3830 (21.1)	12 491 (23.4)		3830 (21.1)	7487 (20.6)	
Missing	177 (1.0)	594 (1.1)		177 (1.0)	325 (0.9)	
Smoking status						
Non‐smoker	6993 (38.6)	22 891 (42.9)	0.09	6993 (38.6)	13 897 (38.3)	0.05
Current/former smoker	9271 (51.1)	25 259 (47.3)		9271 (51.1)	19 090 (52.6)	
Missing	1871 (10.3)	5269 (9.9)		1871 (10.3)	3283 (9.1)	
Barthel index						
100	13 568 (74.8)	40 088 (75.0)	< 0.01	13 568 (74.8)	27 709 (76.4)	0.04
< 100	4251 (23.4)	12 399 (23.2)		4251 (23.4)	8064 (22.2)	
Missing	316 (1.7)	932 (1.7)		316 (1.7)	497 (1.4)	
Type of hospital						
Community hospital	12 802 (70.6)	37 793 (70.7)	< 0.01	12 802 (70.6)	25 759 (71.0)	< 0.01
Academic hospital	5333 (29.4)	15 626 (29.3)		5333 (29.4)	10 511 (29.0)	
Admission year						
2019	8424 (46.5)	26 679 (49.9)	0.07	8424 (46.5)	17 092 (47.1)	0.01
2020	9711 (53.5)	26 740 (50.1)		9711 (53.5)	19 178 (52.9)	
**Comorbidities**						
Charlson comorbidity index						
0–2	10 016 (55.2)	30 508 (57.1)	0.07	10 016 (55.2)	20 320 (56.0)	0.03
3–5	3603 (19.9)	11 000 (20.6)		3603 (19.9)	7139 (19.7)	
≥ 6	4221 (23.3)	10 929 (20.5)		4221 (23.3)	8334 (23.0)	
Missing	295 (1.6)	982 (1.8)		295 (1.6)	477 (1.3)	
Emergency hospitalization	2499 (13.8)	8044 (15.1)	0.04	2499 (13.8)	4809 (13.3)	0.02
Coronary artery disease	3348 (18.5)	15 868 (29.7)	0.27	3348 (18.5)	6458 (17.8)	0.02
Cerebrovascular disease	2689 (14.8)	3891 (7.3)	0.24	2689 (14.8)	5211 (14.4)	0.01
Peripheral artery disease	509 (2.8)	754 (1.4)	0.10	509 (2.8)	917 (2.5)	0.02
Lung cancer	5284 (29.1)	12 654 (23.7)	0.12	5284 (29.1)	10 639 (29.3)	< 0.01
Upper gastrointestinal cancer	1866 (10.3)	3989 (7.5)	0.10	1866 (10.3)	3673 (10.1)	< 0.01
Lower gastrointestinal cancer	1518 (8.4)	3935 (7.4)	0.04	1518 (8.4)	2687 (7.4)	0.04
Leukemia and lymphoma	3268 (18.0)	15 320 (28.7)	0.25	3268 (18.0)	6502 (17.9)	< 0.01
Medications						
Anticoagulants	1645 (9.1)	4173 (7.8)	0.05	1645 (9.1)	2896 (8.0)	0.04
Antibiotics	4057 (22.4)	15 032 (28.1)	0.13	4057 (22.4)	8043 (22.2)	< 0.01

*Note:* Data are presented as *n* (%).

Abbreviation: ASD, absolute standardized difference.

^a^ An ASD > 0.1 is considered imbalanced.

**TABLE 2:** Daily opioid consumption in the matched groups.

	Clopidogrel group	Aspirin group	*p*
Daily opioid consumption (mg/day)^a^	16.4 (5.0–41.5)	21.4 (6.0–61.4)	< 0.001

*Note:* Data are presented as the median (interquartile range).

^a^ Daily opioid consumption is presented as morphine milligram equivalents.

**TABLE 3:** Odds ratios for analgesic use and delirium occurrence in the matched groups.

	Clopidogrel group (*N* = 18 135)	Aspirin group (*N* = 36 270)	Adjusted odds ratio	*p*
Opioid	2039 (11.2%)	4844 (13.4%)	0.83 (0.78–0.88)	< 0.001
Acetaminophen	6952 (38.3%)	14 364 (39.6%)	0.91 (0.88–0.95)	< 0.001
NSAIDs	4573 (25.2%)	10 594 (29.2%)	0.83 (0.80–0.87)	< 0.001
Gabapentinoids	1737 (9.6%)	3630 (10.0%)	0.95 (0.89–1.01)	0.111
14‐day delirium	315 (1.7%)	719 (2.0%)	0.80 (0.69–0.92)	0.002

*Note:* Odds ratios are presented as values (95% confidence intervals) with reference to the aspirin group. Odds ratios were calculated using a multivariable logistic regression model fitted with generalized estimating equations to account for within‐hospital correlations.

Abbreviation: NSAIDs, nonsteroidal anti‐inflammatory drugs.

## 4 Discussion

Our study successfully demonstrated that compared with aspirin, the use of clopidogrel was significantly associated with reduced opioid consumption in cancer patients receiving chemotherapy. These findings suggest that clopidogrel may have unique analgesic properties, particularly in the management of cancer pain. The difference in opioid dose between groups was at most 5 mg and relatively small. However, older and frail patients are at increased risk of delirium, falls, and constipation; therefore, even a modest reduction in opioid use may be beneficial. Conducting a randomized controlled trial to investigate the analgesic effects of clopidogrel presents ethical challenges owing to the associated risk of bleeding complications. Our findings demonstrated the importance of using real‐world evidence to assess drug repositioning for novel therapeutic applications, particularly when conventional clinical trial designs are not feasible [18, 19]. Central sensitisation is considered a possible target for the exploration of novel analgesic agents. An emerging concept of central sensitisation suggests that immune cells, such as microglia, and neurones form an integrated network, where the activation of an immune response modulates the excitability of nociceptive pathways [20]. The P2Y12 receptor is the primary site where nucleotides act to induce microglial activation during the initial response to threatened homeostasis, such as inflammation [21]. Clopidogrel is an irreversible antagonist of the P2Y12 receptor. Once it binds to the P2Y12 receptor, it remains bound and interferes with the intracellular signaling mediated by the receptor.

To date, there are no reports indicating the analgesic potential of clopidogrel in clinical settings, except for our previous small‐sample study [6]. This study presents an example of drug repositioning in which the mechanism of action against a novel therapeutic target was inferred through genetic association analysis, and the practical efficacy of the candidate drug was validated using real‐world data.

In this study, no significant difference was observed between two groups in the proportion of patients receiving gabapentinoids, which are medications used for neuropathic pain. If the analgesic effect of clopidogrel is mediated through spinal microglia‐related pathways, it would be reasonable to expect a reduction in gabapentinoid use in the clopidogrel group. Several explanations may account for this finding. First, a statistical explanation should be considered. As shown in Table 3, the use of gabapentinoids was approximately 10%, which is considerably lower than that of acetaminophen or NSAIDs. Therefore, a type II error due to limited sample size cannot be excluded. Second, in Japan, although gabapentinoids are indicated for neuropathic pain, they are often prescribed in clinical practice for conditions beyond strictly defined neuropathic pain. The Japanese term “shibire” encompasses a broad range of symptoms, including neuropathic pain, hypesthesia, paresthesia, and impaired fine motor skills. Consequently, clinicians may not always strictly differentiate whether the complaint of “shibire” reflects neuropathic pain or hypesthesia when prescribing gabapentinoids. As a result, it is possible that gabapentinoids were continued even after painful CIPN improved with clopidogrel, due to the persistence of residual hypesthesia.

Additionally, there is also a question as to whether clopidogrel reaches the cerebrospinal fluid. Clopidogrel is generally considered to have poor permeability across the intact blood–brain barrier, and there are few reports quantifying cerebrospinal fluid concentrations sufficient to affect spinal microglia after oral administration. In clinical settings, chemotherapy is known to induce sustained neuroinflammation, and persistent neuroinflammation has been reported to increase blood–brain barrier permeability [22, 23]. Furthermore, the blood–spinal cord barrier, which is relevant to spinal microglial activity, is known to be more permeable than the blood–brain barrier [24]. Taken together, in cancer patients undergoing chemotherapy—where chronic neuroinflammation and potential alterations of the blood–brain/spinal cord barriers may occur—the distribution of clopidogrel into the spinal cord compartment may be altered and could potentially reach levels relevant to microglial modulation under certain conditions. However, a possible influence of clopidogrel on microglial activity remains speculative and warrants further investigation.

Our study also found an association between clopidogrel use and lower odds of developing hyperactive delirium. Opioid use is a well‐documented risk factor for delirium, and the reduced opioid consumption observed in the clopidogrel group may partially explain this finding [12]. On the other hand, previous studies have implicated microglial activation in the pathogenesis of delirium [25, 26]. Although these studies did not address the relationship between microglial activation and P2Y12 receptors, it is plausible that P2Y12 receptor antagonists might mitigate the development of delirium by inhibiting microglial activation in the brain.

### 4.1 Limitations

This study had several limitations. First, diagnoses derived from administrative databases tend to be less reliable than those obtained from planned prospective studies. Second, it was not possible to completely eliminate confounding factors. While we conducted propensity score‐matched analyses using 18 potential confounders to balance patient characteristics between the two groups, unmeasured confounders such as pain severity, cancer stage, race, socioeconomic status, education level, and laboratory data may still have influenced the outcomes. Third, defining the clopidogrel and aspirin groups based on antiplatelet use within 2 days of admission excludes patients who start therapy on or after the third day. If the study were designed as an RCT, patients not taking antiplatelets at admission would be excluded; therefore, although this approach reduces the sample size, it is considered reasonable in terms of minimizing the impact of confounding. Fourth, patients with schizophrenia were excluded. Although schizophrenia is a risk factor for delirium, the exclusion accounted for < 1% of patients and is unlikely to have had a substantial impact on the results. Fifth, dementia was not adjusted for between the two groups. Patients with dementia are more likely to receive antipsychotics for nighttime wandering, which may have been captured in the delirium outcome under this study design. However, in clinical practice, distinguishing delirium from dementia is often difficult. It is also unlikely that dementia would have influenced the choice between clopidogrel and aspirin, and therefore it is not expected to have substantially impacted the results. Lastly, the method used in this study to identify hyperactive delirium based on the prescriptions of antipsychotic medications may have lower accuracy than diagnostic approaches that utilize scoring systems [9].

## 5 Conclusions

Among cancer patients receiving inpatient chemotherapy, compared with aspirin use, clopidogrel use was significantly associated with reduced opioid consumption; lower odds of using opioids, acetaminophen, and NSAIDs; and a reduced occurrence of hyperactive delirium. Our findings suggest that clopidogrel may have analgesic effects and additional benefits related to reducing the risk of delirium. Although randomized controlled trials are needed for establishing causal relationships, this study demonstrates the importance of real‐world data in uncovering novel therapeutic uses of existing medications, particularly in contexts where traditional randomized controlled trials are ethically or practically challenging to conduct.

## Funding

This work was supported by the Ministry of Health, Labor and Welfare of Japan (grant numbers 23AA2003 and 24AA2006).

## Ethics Statement

This study was approved by the Institutional Review Board and Ethics Committee of The University of Tokyo (Reference No. 3501‐5; Date: 19 May 2021).

## Consent

Due to the anonymised nature of the data, the requirement for obtaining written informed consent was waived by the Institutional Review Board.

## Conflicts of Interest

The authors declare no conflicts of interest.

## Data Availability

We cannot provide raw data being freely available because we did not obtain agreements to release the data from the supplier and because our ethical approval did not include the release. Instead, the datasets used and/or analyzed during this study are completely available from the corresponding author for collaborative research purposes upon reasonable request.
